# Involvement of MexS and MexEF-OprN in Resistance to Toxic Ion Chelators in *Pseudomonas putida* KT2440

**DOI:** 10.3390/microorganisms8111782

**Published:** 2020-11-14

**Authors:** Tania Henriquez, Tom Baldow, Yat Kei Lo, Dina Weydert, Andreas Brachmann, Heinrich Jung

**Affiliations:** 1 Biozentrum, Mikrobiologie, Ludwig-Maximilians-Universität München, 82152 Martinsried, Germany; T.Henriquez@bio.lmu.de (T.H.); TomBaldow@gmx.de (T.B.); kei.lo@campus.lmu.de (Y.K.L.); Dina.Weydert@campus.lmu.de (D.W.); 2 Biozentrum, Genetik, Ludwig-Maximilians-Universität München, 82152 Martinsried, Germany; brachmann@lmu.de

**Keywords:** *Pseudomonas*, second site revertant, RND transporter, bipyridyls

## Abstract

Bacteria must be able to cope with harsh environments to survive. In Gram-negative bacteria like *Pseudomonas* species, resistance-nodulation-division (RND) transporters contribute to this task by pumping toxic compounds out of cells. Previously, we found that the RND system TtgABC of *Pseudomonas putida* KT2440 confers resistance to toxic metal chelators of the bipyridyl group. Here, we report that the incubation of a *ttgB* mutant in medium containing 2,2’-bipyridyl generated revertant strains able to grow in the presence of this compound. This trait was related to alterations in the pp_2827 locus (homolog of *mexS* in *Pseudomonas aeruginosa*). The deletion and complementation of pp_2827 confirmed the importance of the locus for the revertant phenotype. Furthermore, alteration in the pp_2827 locus stimulated expression of the *mexEF-oprN* operon encoding an RND efflux pump. Deletion and complementation of *mexF* confirmed that the latter system can compensate the growth defect of the *ttgB* mutant in the presence of 2,2’-bipyridyl. To our knowledge, this is the first report on a role of pp_2827 (*mexS*) in the regulation of *mexEF-oprN* in *P. putida* KT2440. The results expand the information about the significance of MexEF-OprN in the stress response of *P. putida* KT2440 and the mechanisms for coping with bipyridyl toxicity.

## 1. Introduction

*Pseudomonas* species are Gram-negative rod-shaped bacteria that exhibit a highly versatile range of activities, including acting as plant protectants (such as *Pseudomonas putida*) or animal pathogens (such as *Pseudomonas aeruginosa*) [1,2]. The bacteria live in changing environments in which they must adapt and respond to stress in order to survive. Resistance-nodulation-division (RND) transport systems contribute to this task by pumping toxic compounds out of cells [3,4]. For example, the expression of *mexXY* is elevated in the presence of ribosome-targeting antibiotics [5], while the *mexCD*-*oprJ* operon is induced by membrane damaging agents [6]. Furthermore, several clinical isolates of *P. aeruginosa* exhibit the so-called *nfxC* phenotype that is characterized by expression of the normally quiescent *mexEF*-*oprN* operon (conferring resistance to quinolones and chloramphenicol), and the downregulation of *oprD* (leading to a decreased uptake of imipenem [7]). The phenotype was originally described in *P. aeruginosa* strain PAO4009 after exposure to norfloxacin [8]). The mutation leading to this phenotype can be located in *mexT* (coding for a regulator of *mexEF-oprN* and *oprD*) [9], *mexS* (regulator of *mexT*) [9], *mexEF-oprN*, *oprD*, *mvaT* [10], *ampR* [11] (the latter two genes code for transcriptional regulators), or in other unknown regions of the genome [12,13]. While most of this information on MexEF-OprN and its regulatory network was derived from clinical isolates of *P. aeruginosa*, little is known about the role and regulation of the RND system in other species, including *P. putida*.

In our previous work, we described that in *P. putida* KT2440 the RND system TtgABC is needed in order to cope with the toxicity of the metal chelating compounds 2,2’-bipyridyl (Bip) and caerulomycin (a 2,2’-bipyridyl-derivaive produced by bacteria and normally found in the environment [14]) [15]. In the absence of a functional transporter, Bip is most likely accumulated inside the cells, where it can sequester copper, iron and other metals, thereby interrupting the normal functioning of metal-dependent enzymes and protein complexes. Indeed, we observed that a *ttgB* mutant (lacking the inner membrane component of the TtgABC system) had an impaired growth phenotype and showed several metabolic defects in the presence of Bip (such as reduced intracellular ATP levels and inhibition of siderophore production) [15]. 

In the present work, we report the generation and characterization of second site revertants of a *ttgB* mutant of *P. putida* KT2440. Revertants were isolated after prolonged incubation of the *ttgB* mutant in presence of Bip and were able to grow in the presence of this compound (like *P. putida* KT2440 wild type strain). The molecular determinants of the phenotype were identified and evaluated by deletion and complementation experiments as well as gene expression analyses. Our results indicate that expression of the genes encoding the RND system MexEF-OprN is responsible for restoring the resistance to Bip in the *ttgB* mutant revertants. Expression of *mexEF*-*oprN* was dependent on alterations in the pp_2827 locus (*mexS*). The results obtained by these experiments provide insights into the role of MexEF-OprN in stress response and reveal components involved in its regulation in *P. putida* KT2440.

## 2. Materials and Methods

### 2.1. Bacterial Strains and Culture Media

A complete list of strains, plasmids, and oligonucleotides used in this research can be found in Table 1 and Table S1. The strains were cultured in King’s Broth (KB) medium [16] at 30 °C and stored as frozen glycerol stocks. 2,2’-Bipyridyl (Bip) (Sigma) was added to the medium at final concentrations of 0.5 or 1 mM when appropriate. For experiments in *P. putida* using plasmids, 1 mg/mL ampicillin or 50 μg/mL kanamycin was used. For susceptibility testing, Mueller Hinton (MH) medium was prepared according to the manufacturer’s instructions.

### 2.2. Colony Morphology Assay

The original protocol from Sakhtah and colleagues [21] was slightly modified. Briefly, overnight cultures were adjusted to an OD_600_ of 3. Then, 10 μL were spotted onto KB agar plates and KB plus 1 mM Bip and incubated at 30 °C for 24 h. The colonies were photographed under visible and UV light. The final image processing was done with ImageJ [22] and Adobe Illustrator.

### 2.3. Generation of Mutants and Complemented Strains

All genes were deleted by homologous recombination using the pNPTS138-R6KT suicide vector [23]. Briefly, upstream and downstream regions of the area to be deleted were amplified and fused by PCR. The resulting amplicon was cloned into pNPTS138-R6KT and used to transform the corresponding initial strain (first recombination). This strain was then grown on cetrimide agar plus 10% sucrose in order to select for the second recombination. The mutant strain was screened by PCR and confirmed by DNA sequencing. For complementation, pp_2827 and *mexF* were amplified by PCR, cloned into the corresponding plasmid (pUCP-Nde and pSEVA224) and used to transform *P. putida* strains. All oligonucleotides used for amplification are listed in Table S1.

### 2.4. Growth Curves

Overnight cultures in KB medium were used to inoculate baffled flasks with 35 mL of KB or KB plus 0.5 mM Bip (initial OD_600_ of ~0.1). The flasks were incubated at 30 °C with continuous shaking at 180 rpm for 8 h. Every 60 min, 1 mL of bacterial culture was taken and used to measure OD_600_. Each experiment was performed a minimum of three times and the data shown in respective graphs represent the average of all replicates. 

### 2.5. Luciferase Activity Assay

A *P_mexF_::luxCDABE* transcriptional reporter gene fusion was generated by PCR amplification of the promoter region of the *mexEF-oprN* operon and cloning of the resulting fragment into the BamHI and XhoI sites of plasmid pBBR1-MSC5-*lux* [20]. *P. putida* strains were transformed with the resulting plasmid pBBR1-MCS5-*P_mex_*::*lux*. Overnight cultures of the resulting strains were used to inoculate 96-well plate in KB with or without 0.5 mM Bip and 30 μg/mL gentamicin. The final volume of the wells was 100 μL with an initial OD_600_ of 0.1. The experiment was performed at 30 °C with continuous shaking for 12 h. After 8 h of growth, luminescence was measured and normalized against the OD_600_ of each strain. Growth and luminescence were measured in a CLARIOstar Plus (BMG LABTECH^®^, Ortenberg, Germany). 

### 2.6. Susceptibility Testing by Diffusion Method

Bacteria were grown on MH agar plates and then used to prepare an inoculum on saline solution (0.85% *w*/*v* NaCl) equivalent to a McFarland 0.5 (OD_625_ of 0.08–0.13). This bacterial suspension was used to inoculate a new MH plate with a cotton swab. The inoculation was made over the plate in three different directions (to get a complete lawn). Then, susceptibility discs from Thermo Scientific™ Oxoid™ were place over the plate using an antimicrobial susceptibility disc dispenser (Thermo Scientific™ Oxoid™, Waltham, MA, USA) and pressed against the agar with tweezers. For this assay, discs of Gentamicin (10 µg) and chloramphenicol (30 µg) were used. Finally, the plates were incubated for 18 h at 30 °C and the inhibition zone around each antibiotic was measured with a ruler. 

### 2.7. Genomic Sequence Analysis

Genomic DNA of revertant and wild type strains was extracted using the Wizard^®^ SV Genomic DNA Purification System (Promega) and for each sample purity was determined on NanoDrop ND-1000 (PeqLab). Library preparation was performed with 100 ng of genomic DNA each, as quantified on Qubit 2.0 Fluorometer (ThermoFisher Scientific with ds HS Assay Kit), using the Nextera DNA Flex Library Prep Kit (Illumina) according to manufacturer’s instructions. Libraries were quality controlled with DNA High Sensitivity DNA Kit on Bioanalyzer (Agilent) and quantified on Qubit 2.0 Fluorometer (ThermoFisher Scientific with ds HS Assay Kit). Genome sequencing was performed in the Genomics Service Unit (LMU Biocenter, Munich, Germany) on Illumina MiSeq with v3 chemistry (2× 250 bp paired-end sequencing). Genome assemblies and variant detection were performed on CLC Genomics Workbench 9 (Qiagen). The data have been deposited with links to BioProject accession number PRJNA670853 in the NCBI BioProject database (https://www.ncbi.nlm.nih.gov/bioproject/).

### 2.8. Statistical Analysis

GraphPad/Prism 8 was used for statistical analysis. One-way ANOVA with Dunnett’s multiple comparison and *t*-test were performed as appropriated. All experiments were performed a minimum of three times.

## 3. Results 

### 3.1. Bip-Driven Generation of Second Site Revertant Strains from the ttgB Mutant

**Figure 1:**
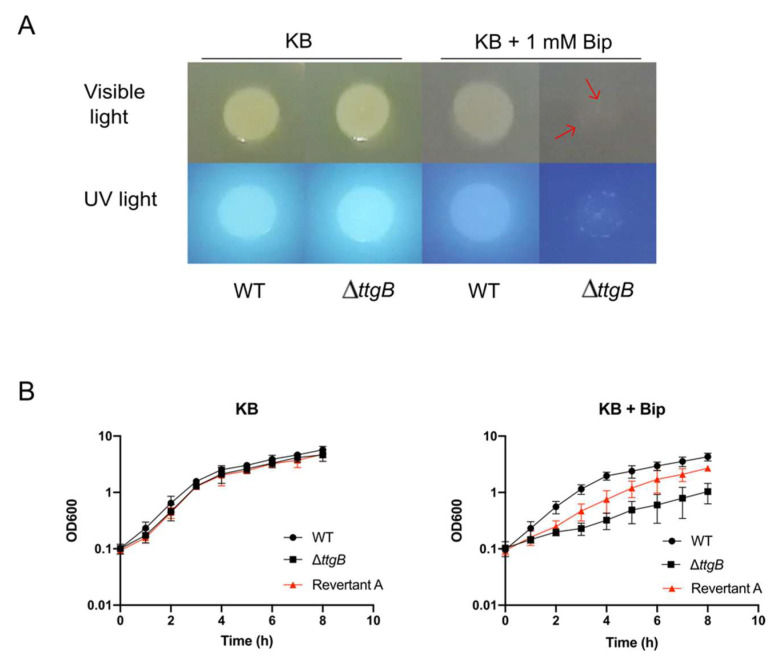
Second site mutation rescues growth of Δ*ttgB* strain in presence of Bip. (**A**). Overnight cultures were spotted onto King’s Broth (KB) agar plates with or without supplementation with 1 mM Bip and incubated at 30 °C. After 24 h, pictures were taken using visible and UV light. The red arrows indicate the position of some of the revertant colonies that are better visible in UV light. (**B**) Growth curves of wild type, Δ*ttgB* and Revertant A strain (red) were performed in KB and KB plus 0.5 mM Bip for 8 h at 30 °C and continuous shaking (180 rpm). One ml of culture was taken and used to record its OD_600_ every 60 min. Experiments were performed a minimum of three times.

In our previous research, we have described that deletion of *ttgB* creates a growth defect in presence of Bip and inhibits the production of the siderophore pyoverdine [15]. This phenotype was most likely related to the accumulation of Bip inside the cell in absence of a functional TtgABC system. In the course of subsequent experiments, we observed that after prolonged incubation in presence of 1 mM Bip, the Δ*ttgB* strain derived from *P. putida* KT2440 started to grow. More specifically, in a colony morphology assay on KB agar plates supplemented with 1 mM Bip, *P. putida* KT2440 (wild type) formed a large colony, while the Δ*ttgB* mutant yielded very small colonies in the inoculated area within 24 h (Figure 1A). Furthermore, when exposed to UV light, wild type colonies and the small colonies derived from the Δ*ttgB* mutant were fluorescent, suggesting that the cells produced the siderophore pyoverdine under the indicated experimental conditions (Figure 1A). We wondered if the small colonies were the result of very slow growth of the mutant or due to compensating mutations elsewhere in the genome. In order to answer this question, bacteria of the small colonies were isolated on KB agar plates without Bip and the colony morphology assay was repeated. Contrary to the original Δ*ttgB* mutant, all isolates formed large colonies in the presence of Bip, similar to wild type. More quantitative analyses of the growth dynamics in liquid KB medium containing 1 mM Bip revealed that the growth of the isolated strains was faster compared to the Δ*ttgB* mutant and even similar to the wild type (Figure 1B). In the absence of Bip, wild type, original Δ*ttgB* mutant and isolated strains grew equally well in KB medium (Figure 1B). Altogether, these results suggest that (an) additional mutation(s) compensate(s) for the deletion of *ttgB* in the isolated strains (called hereafter Revertants A, B and C) and restored the resistance against Bip. 

### 3.2. Alteration in pp_2827 Rescues Growth of the ttgB Mutant in Presence of Bip

To obtain information on the molecular basis of the second site mutation in Revertants A, B and C, a genomic sequence analysis was performed. In Revertant A, a single base substitution was found in locus pp_2827 (A841G), predicted to encode a 340 amino acid oxidoreductase/dehydrogenase [24,25]. The alteration would lead to the substitution of the amino acid isoleucine by valine (I281V) in the putative zinc-binding cassette of the predicted enzyme (Supplementary Material, Figure S1A). In Revertant B, we found an insertion of 17-kb-long transposon Tn*4652* (Tsuda & Iino, 1987) after position 891 in pp_2827, leading to a replacement of the 43 C-terminal amino acids. This fusion also disrupts the putative zinc-binding cassette (Supplementary materials, Figure S1A). In Revertant C, a 250 kb genomic region between pp_3382 and pp_3585 is present in multiple copies (Supplementary Material, Figure S1B). This region is flanked by pp_3381 and pp_3586, two copies of the IS110-like element ISPpu9, which is present 7 times in the *P. putida* KT2440 genome [26]. We focused on Revertant A in subsequent analyses.

**Figure 2:**
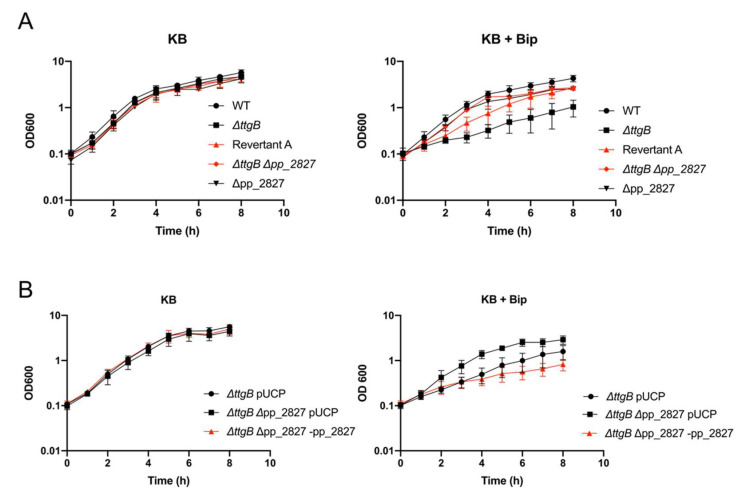
Alteration in the locus pp_2827 affects growth of the Δ*ttgB* mutant in presence of Bip. (**A**) Growth curve of wild type, Δ*ttgB,* Δpp_2827 and strains with revertant phenotype (in red: Revertant A and the engineered Δ*ttgB* Δpp_2827 strain). (**B**) Expression of pp_2827 from plasmid pUCP-pp_2827 reduces growth of the Δ*ttgB* Δpp_2827 strain. For (**A**,**B**), growth curves were recorded in KB and KB plus 0.5 mM Bip at 30 °C and continuous shaking (180 rpm) for 8 h. Every 60 min, 1 mL of culture was taken and used to measure the OD_600_. Experiments were performed a minimum of three times.

To further investigate the role of pp_2827 in the revertant phenotype, we deleted pp_2827 in the Δ*ttgB* mutant. Our results indicated that the growth advantage of the Δ*ttgB* Δpp_2827 strain in presence of Bip was similar to the Revertant A strain (Figure 2A). In parallel, deletion of pp_2827 in the wild type strain did not result in any growth difference (Figure 2A). Obviously, the functional TtgABC system of the wild type was able to cope with Bip toxicity. In accordance with these results, the complementation of pp_2827 in the Δ*ttgB* Δpp_2827 strain made the bacterium susceptible to the toxicity of Bip (Figure 2B). Taken together, these results indicate that alterations in the locus pp_2827 can rescue growth of the *ttgB* mutant in presence of Bip.

### 3.3. Alterations in pp_2827 Confer Bip Resistance by Stimulating Expression of mexEF-oprN 

In order to further explore the resistance mechanism to Bip in Revertant A, we analyzed possible links of pp_2827 to other genes. In *P. aeruginosa*, the locus orthologous to pp_2827 is pa2491 (*mexS*), which together with the regulator gene pa2492 (*mexT*) is located immediately upstream of the *mexEF-oprN* operon (pa2493–pa2495) [27]. The gene products of both *mexS* and *mexT* were previously implicated in the regulation of the expression of *mexEF-oprN* [9,28,29], an RND transporter that is normally quiescent in *P. aeruginosa* [30]. In *P. putida* KT2440, pp_2827 and pp_2826 (*mexT*), and the loci orthologous to *mexEF-oprN* (pp_3425–pp_3427) are located far away from each other in the genome [24,25]. The role of pp_2827 in *P. putida,* to our knowledge, has not been explored yet.

**Figure 3:**
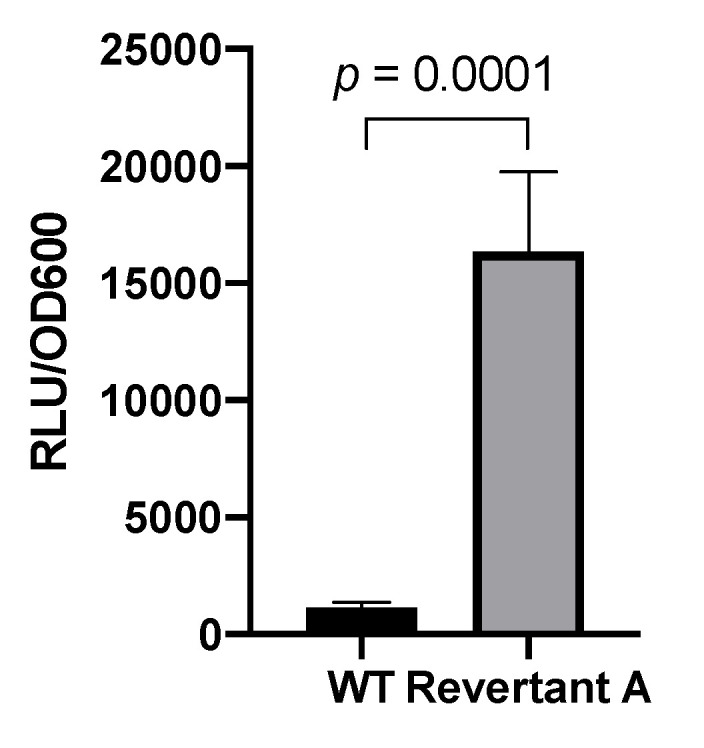
Promoter activity of the *mexEF-oprN* operon in *P. putida* KT2440 in presence of Bip. The transcriptional gene fusion using the *lux* reporter was generated by cloning the promoter region of *mexEF-oprN* into the BBR1-MCS5-*lux* plasmid. For the luciferase assay, overnight cultures of the transformed strains were grown in KB in presence of 0.5 mM Bip and 30 μg/mL gentamicin. After 8 h of growth, the relative light units (RLU) were normalized against the OD_600_ of the cultures. Experiments were performed a minimum of three times.

Thus, we wondered if the observed phenotype in the revertant strains was due to pp_2827 mutation influencing the activity of the MexEF-OprN efflux system. To that end, we analyzed the expression of *mexEF-oprN* in these strains through a transcriptional fusion that contained the promoter of *mexE* fused to the *luxCDABE* operon (*P_mexE_::luxCDABE*) in plasmid pBBR1-MSC5*-lux*. Our results showed that after 8 h of growth, the relative luminescence (normalized against OD_600_) of the Revertant A strain was much higher than the one from the wild type strain (Figure 3), indicating that the *mexEF-oprN* promoter was active. 

**Figure 4:**
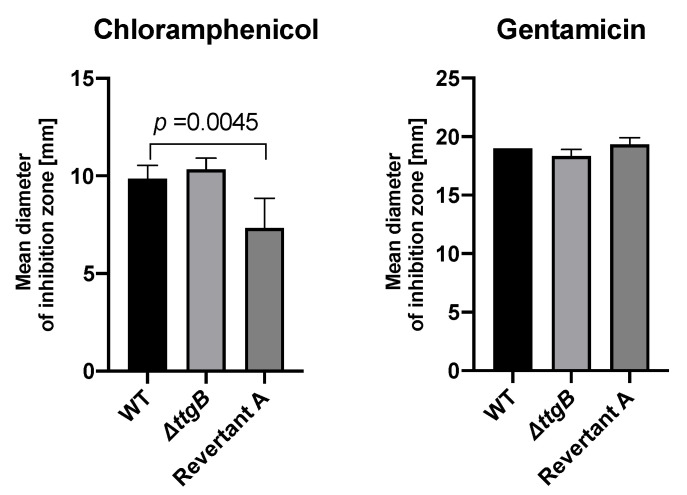
Susceptibility of the Revertant A strain to chloramphenicol. Susceptibility of wild type Δ*ttgB* and Revertant A strains was tested against chloramphenicol in Mueller Hinton (MH) medium through disc diffusion method. Plates were incubated at 30 °C for 18 h, and the halo diameter was measured. Experiments were performed a minimum of three times.

Additionally, the activity of the MexEF-OprN system was indirectly observed through an increased resistance of Revertant A strain to chloramphenicol, while there was no effect in the susceptibility to gentamicin (Figure 4). These results fit to the previously observed MexEF-OprN-mediated resistance pattern of *P. aeruginosa* [31].

**Figure 5:**
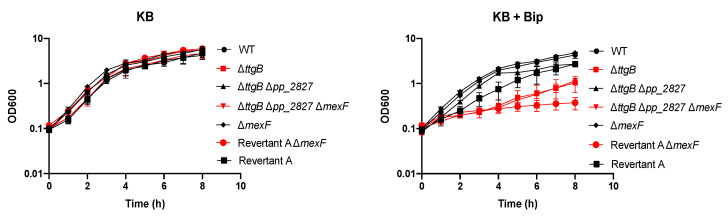
Growth of Δ*ttgB*-derivative strains in presence of Bip is due to MexEF-OprN activity. Bacterial growth was analyzed through growth curves in KB medium supplemented with 0.5 mM Bip at 30 °C with continuous shaking (180 rpm). To that end, overnight cultures were used to inoculate 35 mL of medium (initial OD_600_ of 0.1). Every 60 min, OD_600_ was measured. Strains with phenotype similar to the original Δ*ttgB* strain (Δ*ttgB* Δpp_2827 Δ*mexF* and Revertant A Δ*mexF*) are shown in red. Data are presented as an average of three independent experiments.

In *P. aeruginosa*, the impairment of *mexS*, and the concomitant activation of *mexT*, increases the expression not only of *mexEF-oprN* but also affects other genes (e.g., it represses *oprD*) [32]. Therefore, we wondered whether the Revertant A phenotype in *P. putida* was indeed due to the enhanced expression of the RND system or due to another element regulated by this network. Thus, we deleted the inner membrane component of the RND system, *mexF*, in the Δ*ttgB* Δpp_2827 strain and in Revertant A. Our results showed that both strains lost the resistance to Bip when *mexF* was deleted (Figure 5). On the contrary, in the absence of Bip all strains grew equally well (Figure 5). This indicates that a functional MexEF-OprN system is required for the phenotype of Revertant A. These results were corroborated by complementation experiments, where the expression of *mexF* from a plasmid was able to rescue the growth of the strains similarly as the plasmid-based expression of *ttgB* (Supplementary Materials, Figure S2). Altogether the results indicate that in Revertant A enhanced expression of *mexEF-oprN* is necessary to cope with Bip toxicity in the absence of a functional TtgABC.

### 3.4. The mexEF-oprN Operon is also Upregulated in Revertants B and C

**Figure 6:**
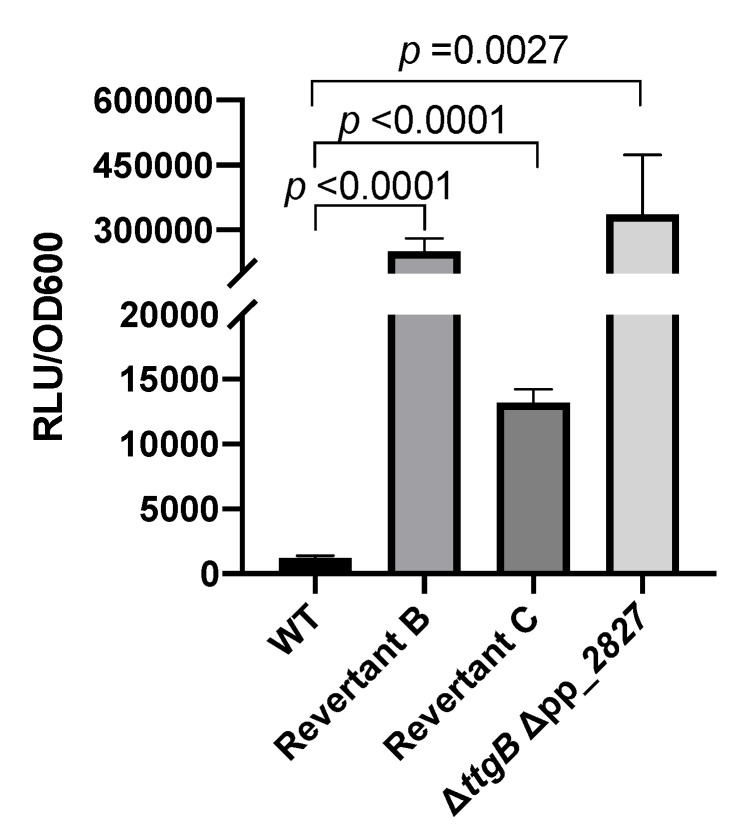
Promoter activity of the *mexEF-oprN* operon in *P. putida* KT2440 in presence of Bip. Overnight cultures of the strains containing the transcriptional fusion of the promoter of *mexEF-oprN* and the *luxCDABE* operon (in plasmid pBBR1-MCS5-*lux*) were grown in KB in presence of 0.5 mM Bip and 30 μg/mL gentamicin. After 8 h, relative light units (RLU) were normalized against the OD_600_. Data are presented as an average of at least three biological replicates.

To obtain more information on the molecular basis of Bip resistance in the other revertant strains, we analyzed expression of the *mexEF-oprN* operon also in Revertant B and C and the engineered Δ*ttgB* Δpp_2827 strain using plasmid pBBR1-MCS5-*P_mex_*::*lux*. Bioluminescence measurements revealed that all three strains expressed *mexEF-oprN*, although to different degrees (Figure 6). The higher expression of *mexEF-oprN* in the Revertant B can also be seen indirectly through the increase in the resistance to chloramphenicol (Figure S3). For Revertant B, upregulation of *mexEF-oprN* was expected since in the strain pp_2827 was altered (transposon insertion) similar to what was observed for Revertant A (point mutation in pp_2827). However, the level of upregulation of the operon was about 20fold higher than in Revertant A and at about the same level as for the engineered Δ*ttgB* Δpp_2827 strain (Figure 6). The mechanism for the moderate upregulation of the *mexEF-oprN* operon in Revertant C remained enigmatic. 

## 4. Discussion

While inactivation of the RND system TtgABC renders *P. putida* KT2440 sensitive to metal ion chelators of the bipyridyl group [15], we describe here that incubation of a *ttgB* mutant of *P. putida* KT2440 in presence of Bip rapidly leads to the generation of second site revertant strains. Since intracellularly accumulated Bip interferes with the cellular homeostasis for iron, cupper and potentially other metal ions required for central metabolic pathways and the respiratory chain, a rapid cellular response compensating the loss of TtgABC activity is not surprising. Known responses to intracellular metal ion limitation in bacteria include the stimulation of the uptake of metal ions, mobilization of limited metal ions from intracellular storage pools, and substitution of metal-dependent enzymes and pathways that function independently of the respective metal ion [33]. None of these possibilities seem to be used by *P. putida* KT2440 under our test conditions. Instead, the primary cause of intracellular ion limitation, the chelator Bip, seems to be removed from cells via an alternative RND system, MexEF-OprN. The latter system is known to be quiescent in *P. aeruginosa* [30], and we showed that the respective operon is also not expressed in *P. putida* KT2440, independent of the presence of Bip in the culture medium. However, in all three revertants generated from the *ttgB* mutant and analyzed here, *mexEF-oprN* was expressed constitutively. Deletion of *mexF* and plasmid-based complementation confirmed that the RND system is indeed required and most likely sufficient for rescuing growth of the Δ*ttgB* strain in the presence of Bip.

Identification of a point mutation and transposon insertion in the locus pp_2827 in the isolated Revertant A and B strains, respectively, and subsequent deletion and complementation analyses with pp_2827 demonstrate that PP_2827 represses the transcription of the *mexEF-oprN* operon. The gene product has been annotated as a zinc-dependent oxidoreductase/alcohol dehydrogenase in the genome of *P. putida* [24,25]. In a previous report, pp_2827 and *mexEF-oprN* were identified as belonging to the PhhR regulon that is made responsible for the activation of genes essential for phenylalanine degradation, phenylalanine homeostasis and other genes of unknown function [34]. However, to the best of our knowledge, this is the first report of an involvement of pp_2827 in the regulation of *mexEF-oprN* expression in *P. putida*. The locus adjacent to pp_2827, pp_2826, encodes a LysR-type transcriptional regulator termed MexT that was previously shown to stimulate *mexEF-oprN* expression in *P. putida* KT2440 [34]. Taken together, the results suggest a regulatory cascade, in which pp_2827 (MexS) inhibits the expression of the *mexEF-oprN* operon by repressing *mexT*. The mechanism of the repression is not known. The putative oxidoreductase MexS is not predicted to contain a DNA binding domain. So far, it can only be speculated that the enzyme produces a metabolite, alters the redox state of another compound or protein, or physically interacts with other proteins leading to repression of *mexT*. In this context, the relatively low levels of *mexEF-oprN* expression observed for Revertant A relative to Revertant B and the engineered Δ*ttgB*Δpp_2827 strain may be due to an only partial inactivation of the putative oxidoreductase (MexS) in Revertant A, while in the latter two strains the enzyme is completely inactive. In fact, the point mutation in Revertant A causes an amino acid substitution (I281V) in the predicted zinc binding domain of the enzyme that may alter enzyme kinetics without abolishing activity. This is the first report showing that an alteration at this site can stimulate *mexEF-oprN* expression. In clinical isolates and laboratory generated spontaneous mutants of *P. aeruginosa* with elevated expression of *mexEF-oprN*, positions such as the N-terminal or the zinc-binding domain were altered in MexS [29,35]. While MexS-dependent regulation of *mexEF-oprN* is dependent of MexT in *P. aeriginaosa* [29], another publication reports that MexS can in principal also have inhibitory effects on gene expression independently of MexT [36]. 

The molecular mechanisms behind the upregulation of *mexEF-oprN* in Revertant C may be due to transposon-based gene duplication events. Our genome comparisons do not hint at changes in the regions of *mexS*, *mexT*, *mexEF-oprN*, *mvaT*, *ampR*, which have been reported to be causative for the *nfxC* phenotype of *P. aeruginosa* [9,10,11]. This result is in accordance with previous reports in clinical strains of *P. aeruginosa*, where significant percentages of the screened strains had a *nfxC* phenotype with unknown mechanisms for the induction of *mexEF-oprN* expression [12]. Furthermore, the relatively low level of expression of *mexEF-oprN* in Revertant C may be explained by a functional pp_2827 (MexS) that most likely inhibits upregulation of the operon. Furthermore, for Revertant C it is not clear whether this upregulation is indeed responsible for Bip resistance. Although the large amplified genomic region (pp_3382 and pp_3585) contains also the *mexEF-oprN* operon, an involvement of other molecular players cannot be excluded.

All three revertant strains analyzed here exhibit a behavior similar to the *nfxC* phenotype of *P. aeruginosa* [12,37], including the expression of *mexEF-oprN* and a higher resistance to chloramphenicol. In the native environment of the soil bacterium *P. putida* KT2440, MexEF-OprN could be involved in the detoxification of natural bipyridyls such as caerulomycin and collismycin that are produced by other bacteria [14,38].

It is interesting to note how quickly (in less than 24 h of exposure) incubation in presence of Bip transformed the *ttgB* mutant into a much more resistant strain (increased resistance to Bip, chloramphenicol and probably other bipyridyls) by second site mutation. Two of the three revertants most likely stem from transposon activity, which at least for Tn*4652* is known to be induced in *P. putida* under stress conditions [39]. From a clinical point of view, this reinforces the necessity to discover effective inhibitors of RND efflux pumps to avoid the fast appearance of spontaneous resistance. Altogether, these results increase the information about the role of MexEF-OprN in stress response in *P. putida* KT2440 and extend the role of MexS in *Pseudomonas* species.

## Figures and Tables

**Table 1:** List of strains and plasmids used in this study.

Name (Strains)	Description	Source
Wild type (WT)	*Pseudomonas putida* KT2440	[17]
Δ*ttgB*	Derived from strain KT2440 by deletion of pp_1385	[15]
Revertant A, B and C	Spontaneous second site revertants of Δ*ttgB* strain	This work
Δ*ttgB* Δpp_2827	Derived from the Δ*ttgB* strain by deletion of pp_2827	This work
Δpp_2827	Derived from strain KT2440 by deletion of pp_2827	This work
Revertant A Δ*mexF*	Derived from Revertant A strain by deletion of pp_3426	This work
Δ*ttgB* Δpp_2827 Δ*mexF*	Derived from the Δ*ttgB* Δpp_2827 strain by deletion of pp_3426	This work
**Name (plasmid)**	**Description**	**Source**
pUCP (pUCP-Nde)	pUCP-NdeI (Amp^R^) shuttle vector	[18]
pUCP-*ttgB*	Derived from pUCP by cloning the *ttgB* gene from wild type strain into the multicloning site	[15]
pUCP-pp_2827	Derived from pUCP by cloning pp_2827 from wild type strain into the multicloning site	This work
pSEVA224	Km^R^; pSEVA221 derivative with *lacI^q^/Ptrc* expression system	[19]
pSEVA224-*mexF*	pSEVA224 derivative with pp_3426 cloned into the multicloning site	This work
pBBR1-MCS5-*lux*	pBBR1-based plasmid containing promoter-less *luxCDABE*, and the *aacC1* gene (Gen^R^)	[20]
pBBR1-MCS5-*P_mex_*::*lux*	pBBR1-MCS5-lux derivative containing *P_mexE_::luxCDABE*	This work

## Supplementary Materials

The following are available online at https://www.mdpi.com/2076-2607/8/11/1782/s1, Figure S1: Mutations in Revertant A, B and C strains, Figure S2: Phenotype complementation with *ttgB* and *mexF* in the Revertant A Δ*mexF* and Δ*ttgB* Δpp_2827 Δ*mexF* strains, Figure S3: Susceptibility of Revertant B to chloramphenicol, Table S1: List of primers used in this study.
