# Supplementary material for: Involvement of MexS and MexEF-OprN in Resistance to Toxic Ion Chelators in Pseudomonas putida KT2440
Source: Microorganisms. 2020 Nov 14;8(11):1782. doi: 10.3390/microorganisms8111782 (PMC7697342; doi:10.3390/microorganisms8111782)
Supplement: Supplementary file 1 [file microorganisms-08-01782-s001.pdf]

Supplementary Material

Article

Involvement of MexS and MexEF-OprN in resistance to toxic ion chelators in *Pseudomonas putida* KT2440

Tania Henriquez[1], Tom Baldow[1], Yat Kei Lo[1], Dina Weydert[1], Andreas Brachmann[2], Heinrich Jung[1]*

[1] Ludwig-Maximilians-Universität München, Biozentrum, Mikrobiologie, Martinsried, Germany
[2] Ludwig-Maximilians-Universität München, Biozentrum, Genetik, Martinsried, Germany
* Correspondence: hjung@lmu.de

Keywords: Pseudomonas; second site revertant; RND transporter; bipyridyls

This PDF file includes:

Tables S1
Figure S1 to S3

Table S1. List of primers used in this study

Name	Sequence (5'- 3')	Description
pUCP-NdeI-MCS-RII	TCATTAATGCAGCTGGCAC	Primer for sequencing MCS of pUCP-NdeI
M13 Fw(-41)	GGTTTTCCCAGTCACGAC	Primer for sequencing MCS of pSEVA224
M13uni	TGTAAAACGACGGCCAGT	Universal primer for plasmid pNPTS138-R6KT and pUCP-NdeI
M13reverse	AACAGCTATGACCATG	Universal primer for plasmid pNPTS138-R6KT and pUCP-NdeI
delmexF_1S	GTCATGGACAAGGACAA	To delete *mexF*
delmexF_1A	AGATGTTGTTGGGGTATTCGCTGAT	To delete *mexF*
delmexF_2S	CGAATACCCCAACAACATCTTCACC	To delete *mexF*
delmexF_2A	CACATCGCTGCTCAATTG	To delete *mexF*
Check mex2R	CCTTGGCAAGAAGTTTGTGCT	To check *mexF* deletion
Check mex2F	TCGAACTGCTTCCACCACA	To check *mexF* deletion
Del_2827_A_s	AATCTTAAGGCCAAAGGCTGTTACC	To delete pp_2827
Del_2827_A_as	GGTACACCTTATATCATCGACCCCT	To delete pp_2827
Del_2827_B_s	TCGATGATATAAGGTGTACCCGTTC	To delete pp_2827
Del_2827_B_as	CCAAACTTACCTAGAAGAAATTTCC	To delete pp_2827
Check_Del_2827_as	AATAGCAGTCACGCAATCACCTCAT	To check pp_2827 deletion
Check_Del_2827_s	AGCAGGTTGAGGTCTACACGACGCA	To check pp_2827 deletion
Clon_pp2827_F	AAAGGCGAGGTATCATATGTCCCG	To clone pp_2827 into pUCP-NdeI
Clon_pp2827_R	TTGTGCCAGGATCCGTCAGTGTTG	To clone pp_2827 into pUCP-NdeI
F-Clone mexF New	GCTCTAGACTACCGATGAACTTCTCG	To clone pp_*mexF* into pSEVA224
R-Clone mexF New	CGAAGCTTGTTCATGCGTGGTTC	To clone pp_*mexF* into pSEVA224
PmexE R	GGACTCGAGCGCCAATGTATTAGT	To clone promoter *mexE* into pBBR1-MCS5-lux
PmexE F2	CGCGGATCCTGAAGCCCACAC	To clone promoter *mexE* into pBBR1-MCS5-lux

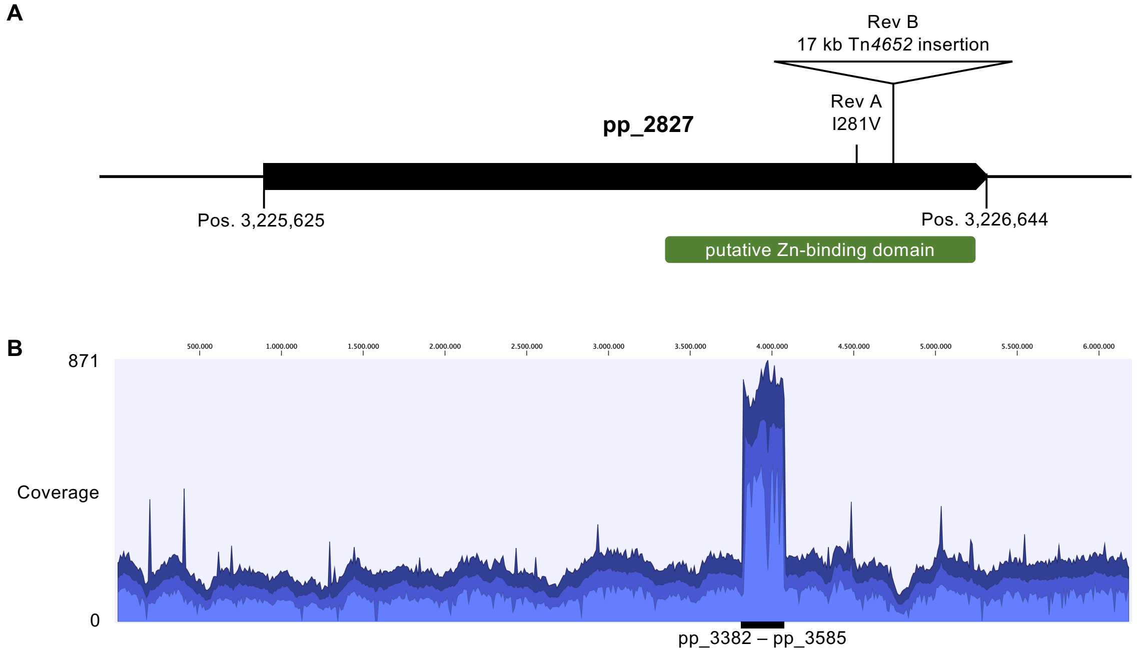

Figure S1. Mutations in Revertant A, B and C strains. (A) Single base substitution in Revertant A and transposon insertion in Revertant B. Positions are given relative to *P. putida* KT2440 reference genome NC_002947.4. (B) Coverage analysis from genomic sequencing of Revertant C showing amplification event in region pp_3382 to pp_3585. Coverage is presented as minimum, average and maximum in sliding 10 kb windows.

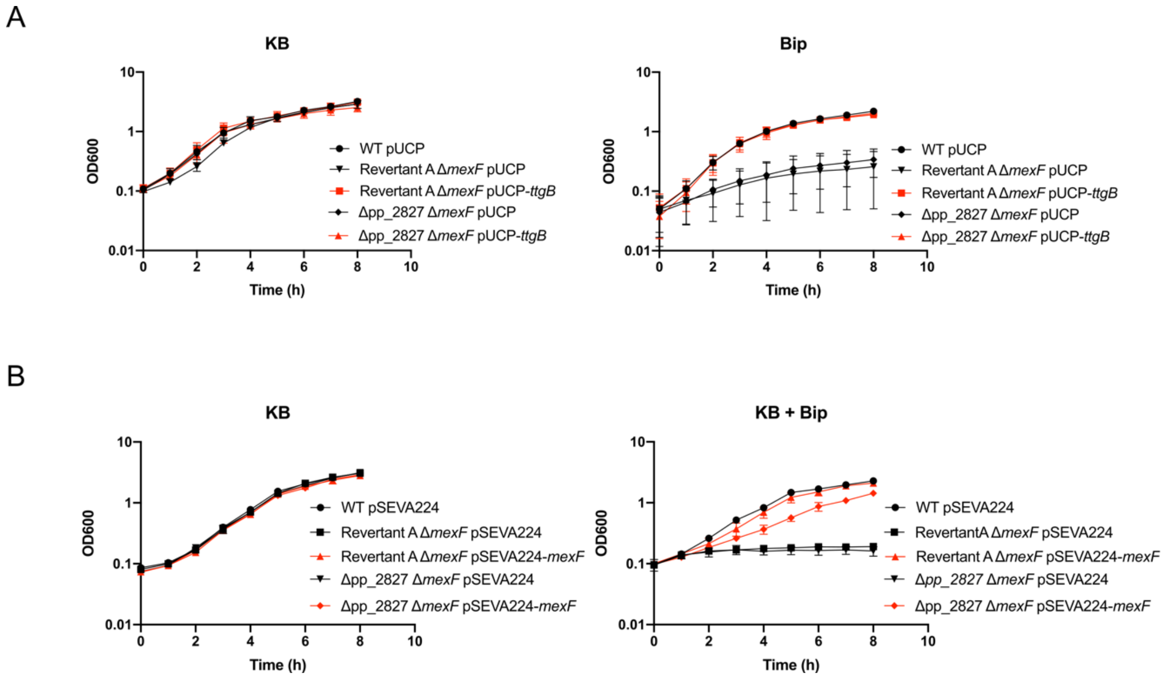

Figure S2. Phenotype complementation with *ttgB* and *mexF* in the Revertant A Δ*mexF* and Δ*ttgB* Δpp_2827 Δ*mexF* strains. Growth curve of wild type, mutants and complemented strains with *ttgB* (A) or *mexF* (B) in KB with or without supplementation. For (A) and (B), growth curves were performed for 8 hours at 30°C and continuous shaking (180 rpm). Every 60 minutes, 1ml of culture was taken and used to measure the OD_{600}. Experiments were performed a minimum of three times.

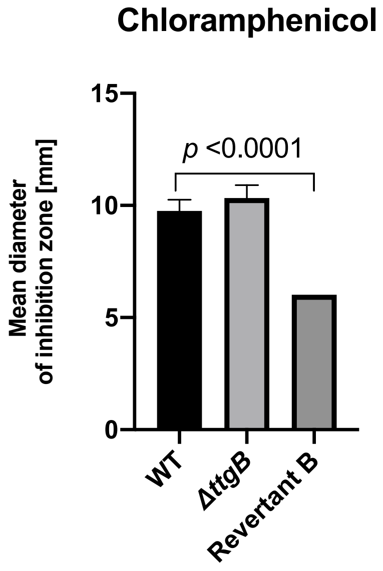

Figure S3. Susceptibility of Revertant B to chloramphenicol. Susceptibility of wild type, Δ*ttgB* and Revertant B strains was tested against chloramphenicol in MH medium through disc diffusion method. After 18 hours of incubation at 30°C, the halo diameter was measured. Data are presented as an average of three independent experiments.
